# The Winter-Type Allele of *HvCEN* Is Associated With Earliness Without Severe Yield Penalty in Icelandic Spring Barley (*Hordeum vulgare* L.)

**DOI:** 10.3389/fpls.2021.720238

**Published:** 2021-09-24

**Authors:** Magnus Göransson, Thorbjörg Helga Sigurdardottir, Morten Lillemo, Therése Bengtsson, Jon Hallsteinn Hallsson

**Affiliations:** ^1^Faculty of Agriculture, Agricultural University of Iceland, Reykjavík, Iceland; ^2^Department of Plant Science, Norwegian University of Life Sciences, Ås, Norway; ^3^Department of Plant Breeding, Swedish University of Agricultural Sciences, Lomma, Sweden

**Keywords:** barley, flowering time, yield, adaptation, *HvCEN*, *Ppd-H1*, *HvFT1*, *HvELF3*

## Abstract

Icelandic barley genotypes have shown extreme earliness both in flowering and maturity compared to other north European genotypes, whereas earliness is a key trait in adapting barley to northern latitudes. Four genes were partially re-sequenced, which are *Ppd-H1, HvCEN, HvELF3*, and *HvFT1*, to better understand the mechanisms underlying this observed earliness. These genes are all known to play a part in the photoperiod response. The objective of this study is to correlate allelic diversity with flowering time and yield data from Icelandic field trials. The resequencing identified two to three alleles at each locus which resulted in 12 haplotype combinations. One haplotype combination containing the winter-type allele of *Ppd-H1* correlated with extreme earliness, however, with a severe yield penalty. A winter-type allele in *HvCEN* in four genotypes correlated with earliness combined with high yield. Our results open the possibility of marker-assisted pyramiding as a rapid way to develop varieties with a shortened time from sowing to flowering under the extreme Icelandic growing conditions and possibly in other arctic or sub-arctic regions.

## Introduction

Barley has proven to be well-suited in harsh climates since its early domestication and is by far the most important cereal crop in Iceland and many other northern and arid regions of the world (Schoppach et al., 2017). Barley is thought to have moved gradually northwards from the center of early domestication and finally been introduced to Scandinavia around 2,000 BCE (Bogucki, 2000), approximately 2,000 years after the first farmers arrived in northern Europe (Skoglund et al., 2012). This lag might be explained in part by a lack of genetic variants suiting the special conditions in northern Europe, such as the shorter growing period and longer days. Sub-arctic agriculture is still at the margin of barley cultivation due to its characteristics such as short and cool growth period, occasional very strong winds, risk of frost in both late spring and early autumn, and a long photoperiod. This is highlighted by the relatively short history of barley cultivation in Iceland wherein barley has been continuously grown in the country since 1923 (Hermannsson, 1993). This is further accentuated by the high variation in yield and dry matter weight experienced by Icelandic farmers (Hilmarsson et al., 2017).

Despite the short history of barley cultivation in Iceland, it is being grown today at around 5,000 ha with an average yield of 3.2 t ha^−1^ (Hilmarsson et al., 2017), which is comparable to similar latitudes in Scandinavia. For example, the yield has been reported at 3.5 t ha^−1^ in the Trøndelag region in Norway (Lillemo et al., 2010). Several possible non-exclusive explanations for the increased barley cultivation in Iceland have been raised, which include favorable climate change, increased testing of Nordic breeding material leading to better selections of varieties, and a local breeding project aimed at breeding varieties especially adapted to the Icelandic climate (Hilmarsson et al., 2017). The Icelandic barley breeding program was initiated in 1990 and has mostly used Nordic material, focusing on adapting the highest yielding cultivars to local conditions by altering their flowering time and improving lodging and wind resistance. The study of Hilmarsson et al. (2017) recently described that the most promising breeding lines have been in field trials as a part of the breeding program together with foreign cultivars in four to five locations around the country every year.

The temperature during summer in Iceland is considerably lower than in other comparable latitudes. It has an average accumulated heat sum over the growth season of 1,300-day degrees (Ólafsson et al., 2007) compared to the Trøndelag region, which has an average heat sum of 1,790-day degrees (Hole and Rafoss, 2010). The study of Mølmann et al. (2021) reviewed how the uniquely long summer days in sub-arctic latitudes can compensate for below-optimal temperature during the growth season for biomass production in forage grasses. It seems that the phenological development of barley under these unique conditions is also different from that observed under higher temperatures (Göransson et al., 2021).

It is necessary to identify the specific alleles underlying traits to obtain the desired breeding targets with the least amount of breeding effort and to understand the interactions between different alleles and genes under different climatic conditions. Underlying traits include earliness, lodging resistance, straw strength, and frost tolerance.

Climate change is predicted to lead to increased temperatures, which will be more pronounced in the sub-arctic region than what is expected on average in the other regions globally (IPCC, 2018). With climate change coinciding with the need to increase global food production to keep up with the rising population, it is important to find ways to diversify the grassland-based agriculture commonly practiced in northern latitudes and to respond to increased droughts in the more arid parts of the world. Many of the globally important grain-producing regions are expected to suffer from severe drought and heat stress, which most likely will have a negative impact on the yield in these regions (Lobell et al., 2008). It is likely that other areas at comparable latitudes (and photoperiods) to Iceland will open as potential barley production regions in the near future based on predictions about future climate scenarios by IPCC (2018). The early flowering Icelandic breeding lines could be a valuable resource for developing high-yielding cultivars for wider distribution in northern latitudes, given that the early flowering traits can be efficiently introgressed into elite barley varieties more suitable to the expected higher average temperature. Under increased aridification, wild relatives of both wheat (*Triticum dicoccoides*) and barley (*Hordeum vulgare* ssp. *spontaneum*) have been shown to respond to climate change by displaying increased earliness (Nevo et al., 2012). This implies that even barley cultivation in more southern locations could benefit from increased understanding of earliness genes from Nordic breeding populations.

The transition from the vegetative state to flowering is controlled by a complex molecular system well-conserved between even distantly related plant species (Blümel et al., 2015; Hill and Li, 2016). Wherein the model organism *Arabidopsis thaliana* has served as an important reference for identification and verification of flowering-related genes (Andrés and Coupland, 2012). Several genes associated with the transition from vegetative growth to flowering in barley have been described and corresponding markers developed, e.g., Cockram et al. (2007), Faure et al. (2012), Varshney et al. (2007), and Wang et al. (2010). In the study of Zakhrabekova et al. (2012), they described a 4 bp deletion in the *HvELF3* gene (syn. *EAM8, mat-a*; orthologous to *ELF3* in *Arabidopsis thaliana*) in the two-row *cv*. “Mari” and suggested that this deletion might explain its earliness and day length neutrality. It also combined with its resistance to lodging allowing the extension of two-row barley into Northern Scandinavia and Iceland. This and similar studies enable the screening of breeding lines with allele-specific markers further opening the door to marker-assisted selection (MAS), a rapid method for introgressing specific trait-related alleles into selected lines (Muthu et al., 2020).

The effect of flowering genes on yield in barley has been reported from regions such as Jordan, where the day length sensitive allele of the *Ppd-H1* was associated with a 30% yield increase compared to the insensitive *ppd-H1* allele (Wiegmann et al., 2019). In northern Europe, the situation is reversed, and the non-responsive *ppd-H1* allele has been associated with increased yield (Wiegmann et al., 2019), highlighting the adaptive importance of *Ppd-H1*. *HvCEN* has been associated with yield and yield-related traits in studies in Australia where yield was positively associated with a QTL very near *HvCEN* (Obsa et al., 2017). *HvCEN* was the peak marker associated with yield in a spring-type × winter-type population tried in Mediterranean conditions, and they reported early heading without yield penalty (Tondelli et al., 2014). In all, 13 haplotypes of *HvCEN* have been described worldwide, out of which types I, II, and III are the most prevalent (Fernandez-Calleja et al., 2021). In the last exon, an SNP codes the amino acid Ala135 in types I and III whereas in type II it codes Pro135 (Comadran et al., 2012). The Ala135 coding haplotype is primarily found in European spring barley and is beneficial in long and cool growth seasons, where it delays flowering to allow for full utilization of the growth season. The Pro135 coding haplotype is beneficial in Mediterranean conditions where early flowering is beneficial to develop kernels before the summer heat (Comadran et al., 2012; Fernandez-Calleja et al., 2021). Sharma et al. (2018) found allelic diversity in *HvFT1* originating from wild barley, which had a reducing effect on yield. Similarly, Wiegmann et al. (2019) found the recessive wild “winter type” allele to reduce yield in a drought-stressed environment. HvELF3 has a defined role in the flowering pathway by downregulating the HvFT1 gene (Boden et al., 2014) but its effect on yield-related characters is less defined.

Implementation of MAS in the breeding program will speed up the selection process, in particular for traits controlled by single or few genes, and add precision by enabling finesses that are difficult to control by traditional breeding such as pyramiding of multiple genes interacting on the same trait (Muthu et al., 2020). Establishing knowledge of available allelic diversity for example for earliness genes by applying allele-specific markers on the Icelandic and Scandinavian barley breeding material will provide the foundation for more precision in the future breeding program.

Two studies on the genetic mechanisms behind the Icelandic early barley have been published. The study of Göransson et al. (2019) identified allelic differences between single nucleotide polymorphism (SNP) markers at the *HvPph-H1*, and *HvFT1* loci as a partial explanation of the extreme earliness in the lines “247-1” and “247-11” observed in multi-location field trials ranging from Bavaria, Germany in the south to Iceland in the north. Göransson et al. (2021) identified variation at *HvELF3, Ppd-H1*, and *HvFT1* as contributing to early maturity in Icelandic barley, in an experiment with contrasting day length and temperature combinations in controlled environments. Comadran et al. (2012) highlighted the contribution of allelic variation at *HvCEN* as a factor to enable latitudinal range extension of barley and recently, whereas Bustos-Korts et al. (2019) and Jayakodi et al. (2020) have reported variation at the *HvCEN* locus as contributing to early flowering.

With this as a background, the objectives of the present study were to re-sequence the four earliness loci *Ppd-H1, HvFT1, HvELF3*, and *HvCEN* in a set of early Icelandic barley lines and their progenitors to elucidate the allelic variation behind the extreme earliness, which has been especially pronounced in lines “247-1” and “247-11”.

## Materials and Methods

### Icelandic Field Tests and Material Selected for Sequencing

#### Agronomic Data

Twenty barley genotypes (Table 1) were selected by analyzing data from geographically dispersed Icelandic cultivar trials between the years 1987 and 2014 (Hilmarsson et al., 2017). The selection was made to include (1) previously known extremely early lines (“247-11” and “247-1”) (Göransson et al., 2019, 2021), (2) selected ancestors based on pedigree data, and (3) cultivars and breeding lines with a high yielding capacity in Iceland. The panel included Icelandic cultivars and breeding lines as well as foreign cultivars that had been tried in a minimum of four trials with yield data. The traits analyzed were yield (t ha^−1^) and heading (number of days from sowing to heading). The heading was scored as the number of days from sowing until half of the plants in the plot had reached Zadoks growth stage 55 (Zadoks et al., 1974). This comparison included 1,608 data points for “yield” (ranging from four to 332 data points for different genotypes) and 814 data points for “heading” (ranging from two to 200 data points for different genotypes). Due to the highly unbalanced data set, best linear unbiased estimates (BLUEs) for heading and yield were calculated using the least square means (LS Means) model with package lme4 in R (Bates et al., 2015). The data were analyzed in a single stage (Piepho et al., 2012; Bernal-Vasquez et al., 2014) using genotype, location, and year as fixed effects whereas replication was a random effect nested within location × year. Based on the BLUEs for heading and yield, an index was calculated by dividing Yield with Heading (DDY) to estimate the lines with the highest yielding capacity and the shortest heading time. The significance between DDY was calculated with Tukey's test with grouping by haplotype combination using package dplyr in R (Wickham et al., 2021).

**Table 1:** Genotypes with row type information, and their haplotypes of four re-sequenced flowering genes: *HvELF3* (blue), *Ppd-H1* (orange), *HvCEN* (green), and *HvFT1* (grey), the geographic origin, release year, and note why they were included in the study.

	**Genotype [row type]**	***HvELF3***	***Ppd-H1***	***HvCEN***	***HvFT1***	**Origin**	**Released**	**Note**
H01	**250-4** [6r]	**E2**	**P2**	**C2**	**F2**	Iceland	Breeding line	High yield
	**Arve** [6r]	**E2**	**P2**	**C2**	**F2**	Norway	1990	In pedigree
	Åsa [6r]	**E2**	**P2**	**C2**	**F2**	Sweden	1942	In pedigree
	Asplund [6r]	**E2**	**P2**	**C2**	**F2**	Sweden	1910	In pedigree
	Maskin [6r]	**E2**	**P2**	**C2**	**F2**	Norway	1918	In pedigree
	Scots Bere [6r]	**E2**	**P2**	**C2**	**F2**	Scotland	Landrace	
	Sigur-F [6r]	**E2**	**P2**	**C2**	**F2**	Faroe Isl.	Landrace	In pedigree
	Tammi [6r]	**E2**	**P2**	**C2**	**F2**	Finland	1937	In pedigree
	Tampa [6r]	**E2**	**P2**	**C2**	**F2**	Faroe Isl.	Landrace	In pedigree
	Varde [6r]	**E2**	**P2**	**C2**	**F2**	Norway	1941	In pedigree
	Vigdis [6r]	**E2**	**P2**	**C2**	**F2**	Norway	1964	In pedigree
	**VoH2825** [6r]	**E2**	**P2**	**C2**	**F2**	Norway	Breeding line	In pedigree
H02	**263-9** [2r]	**E2**	**P2**	**C2**	**F3**	Iceland	Breeding line	High yield
	Binder [2r]	**E2**	**P2**	**C2**	**F3**	Denmark	1913	In pedigree
	Gull [2r]	**E2**	**P2**	**C2**	**F3**	Sweden	1913	In pedigree
	**Kannas** [2r]	**E2**	**P2**	**C2**	**F3**	Sweden	2013	High yield
	**Kria** [2r]	**E2**	**P2**	**C2**	**F3**	Iceland	2005	In pedigree
	Monte-Christo [6r]	**E2**	**P2**	**C2**	**F3**	India	Landrace	In pedigree
	**Skegla** [2r]	**E2**	**P2**	**C2**	**F3**	Iceland	2002	In pedigree
H03	Akka [2r]	**E1**	**P2**	**C2**	**F1**	Sweden	1970	
	Arla [2r]	**E1**	**P2**	**C2**	**F1**	Sweden	1962	
	Deba abed [2r]	**E1**	**P2**	**C2**	**F1**	Denmark	1964	In pedigree
	**Minttu** [2r]	**E1**	**P2**	**C2**	**F1**	Finland	2005	High yield
	Triumph [2r]	**E1**	**P2**	**C2**	**F1**	Germany	1973	In pedigree
H04	**Is-046** [2r]	**E3**	**P2**	**C2**	**F1**	Iceland	Breeding line	In pedigree - DLN
	**Mari** [2r]	**E3**	**P2**	**C2**	**F1**	Sweden	1960	In pedigree - DLN
	**Teista** [2r]	**E3**	**P2**	**C2**	**F1**	Iceland	2021	In pedigree - DLN
H05	Opal [2r]	**E2**	**P2**	**C2**	**F1**	Denmark	1922	In pedigree
	**Pernilla** [2r]	**E2**	**P2**	**C2**	**F1**	Sweden	1979	In pedigree
	Swallow [2r]	**E2**	**P2**	**C2**	**F1**	Germany	1962	In pedigree
H06	**06-130** [6r]	**E2**	**P2**	**C1**	**F1**	Iceland	Breeding line	High yield
	**292-51** [6r]	**E2**	**P2**	**C1**	**F1**	Iceland	Breeding line	High yield
H07	**292-2** [6r]	**E2**	**P2**	**C1**	**F2**	Iceland	Breeding line	High yield
	**Wolmari** [6r]	**E2**	**P2**	**C1**	**F2**	Finland	2010	In pedigree
H08	**247-1** [6r]	**E2**	**P1**	**C2**	**F2**	Iceland	Breeding line	Extreme Earliness
	**247-11** [6r]	**E2**	**P1**	**C2**	**F2**	Iceland	Breeding line	Extreme Earliness
H09	Fimbul [6r]	**E1**	**P1**	**C1**	F1*	Sweden	1946	In pedigree
H10	Golf [2r]	**E1**	**P3**	**C2**	**F1**	UK	1983	In pedigree
H11	**Nairn** [2r]	**E2**	**P1**	**C2**	**F1**	Scotland	1984	In pedigree
H12	**Saana** [2r]	**E3**	**P2**	**C2**	**F2**	Finland	1996	High yield

**Figure 1:**
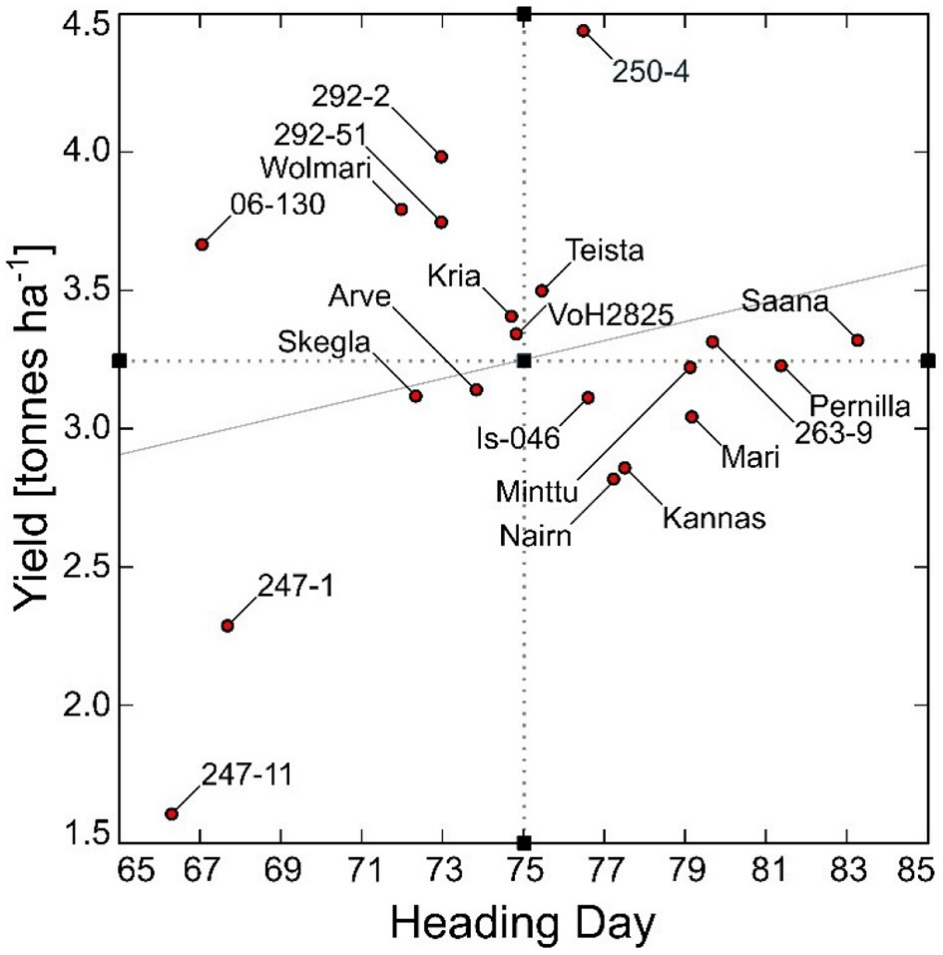
Yield (t ha^−1^) and heading day (days from sowing to heading) for 20 barley genotypes under Icelandic environmental conditions for the period 1987–2014. The mean values for heading day and yield are indicated as black boxes on the x- and y-axes, respectively, and the trendline is indicated as the gray slope.

*The 20 genotypes marked in bold indicate that heading day and yield data have been analyzed (see Figure 1). H01–H12 indicate haplotype combinations. DLN indicates day-length neutral lines identified in Göransson et al. (2021)*.

#### Resequencing Panel

Twenty additional genotypes were included for resequencing based on their role as parental lines to selected barley varieties or breeding lines of interest, which resulted in a panel of 40 genotypes (Table 1). The pedigree of the Icelandic breeding lines with their ancestors back to the relevant landraces, wherever possible, was compiled based on resources such as logbooks, information from breeding companies and gene banks, official cultivar registration data, and scientific publications, e.g., Manninen and Nissilä (1997) and Nurminiemi et al. (1996). This allowed the selection of the relevant ancestors for sequencing (Supplementary Figure 1). Among the 40 genotypes, 20 were two-rowed and 20 were six-rowed, 39 were characterized as spring barley, and the winter barley cultivar “Fimbul” was included based on pedigree data.

### DNA Isolation and PCR Amplification

Deoxyribonucleic acid was isolated using the NucleoSpin^®^ Plant II kit from Macherey-Nagel, Dueren, Germany (http://www.mn-net.com/) following the protocol of the manufacturer. PCR primers were designed based on sequences available for the “Morex” *HvELF3* (syn. *Eam8, Mat-a*; NCBI GenBank accession number JN180296), *Ppd-H1* (GenBank accession number AY943294), *HvCEN* (syn. *Eps2s, eam6;* GenBank accession number JX648182), and *HvFT1* (syn. *Vrn3;* GenBank accession number EU007831). The varieties selected were genotyped for the “Mari” deletion through sequencing of a 200 bp PCR fragment from exon 2, amplified using primers *HvELF3_delMariF1* and *HvELF3_delMariR1* (nucleotide sequences deposited with NCBI GenBank under accession numbers MZ286789-MZ286828). There is a total of 3,128 bp from the *Ppd-H1* gene, which were amplified and sequenced using seven primer pairs, covering exons 1–8 (nucleotide sequences deposited with NCBI GenBank under accession numbers MZ286829-MZ286868). From the *HvCEN* gene, 1,001 bp were amplified and sequenced using three primer pairs (nucleotide sequences deposited with NCBI GenBank under accession numbers MZ286869-MZ286908), and 2,595 bp were amplified and sequenced using six primer pairs from the *HvFT1* gene (nucleotide sequences deposited with NCBI GenBank under accession numbers MZ286909-MZ286948). Same primers were used for PCR and sequencing, all listed in Table 2 with information on sequence and annealing temperature. PCR products were purified using a NuceloSpin^®^ PCR clean-up Gel extraction kit from Macherey-Nagel according to the recommendations of the manufacturer.

**Table 2:** Primer names and sequences used for re-sequencing four flowering genes, along with their annealing temperature (Tm).

**Primer name**	**Gene**	**Sequence (5^**′**^-3^**′**^)**	**Tm**
Hvul_PpdH1-F1	Ppd-H1	5′-CGACTGTCATTCACGGCC-3′	58.5
Hvul_PpdH1-F2	Ppd-H1	5′-TGCTGTTGCTGCTGGCTC-3′	58.2
Hvul_PpdH1-F3	Ppd-H1	5′-TTGTTTGGACTTTGGATAAACTTG-3′	55.9
Hvul_PpdH1-F4	Ppd-H1	5′-AGAATACTTACATGTGTGAGAAGT-3′	55.9
Hvul_PpdH1-F5	Ppd-H1	5′-GCAAAGCATAATATCAGTGTCCT-3′	57.1
Hvul_PpdH1-R1	Ppd-H1	5′-GTAGCAGTATACCTTAAGTACA-3′	54.7
Hvul_PpdH1-R2	Ppd-H1	5′-AGCTTCCTTATCCTAACAATTGT-3′	55.3
Hvul_PpdH1-R3	Ppd-H1	5′-ACGGATGATTTCAGGATTCAC-3′	55.9
Hvul_PpdH1-R4	Ppd-H1	5′-GTACTAGGTATAGCTAGGTGCG-3′	60.3
Hvul_PpdH1-R5	Ppd-H1	5′-ACAAGAATCAGCTGTCTAATTAGT-3′	55.9
Hvul_PpdH1-F6	Ppd-H1	5′-TCCAACCCCACTCGCCG-3′	60.0
Hvul_PpdH1-R6	Ppd-H1	5′-ACAAGATAAGTATTGGTGGAGC-3′	56.5
Hvul_PpdH1-F7	Ppd-H1	5′-CTCAAGTGCCCAACCAGC-3′	58.2
Hvul_PpdH1-R7	Ppd-H1	5′-GGAACTTAATCAATACGAAGTGG-3′	57.1
Hvul_PpdH1-F8	Ppd-H1	5′-CCAGTGTTGTCAATCCTTCGG-3′	59.8
Hvul_PpdH1-R8	Ppd-H1	5′-CTGAATGAGTTGCTACCATAGTTGG-3′	61.3
Hvul-PpdHI-R9	Ppd-H1	5′-GAGACGCGGAATTTTATTTAAC-3′	54.7
Hvul_PpdH1-R1b	Ppd-H1	5′-TGTCTGAAAATATTACAGGTAGC-3′	55.3
Hvul_PpdH1-F9	Ppd-H1	5′-ATAATGGCAGTGGCACTC-3′	53.7
Hvul_PpdH1-R9b	Ppd-H1	5′-GACTGATCCGGAGACATG-3′	55.9
Hvul_PpdH1-F10	Ppd-H1	5′-TTGTCAATCCTTCGGGTC-3′	53.7
Hvul_PpdH1-R10	Ppd-H1	5′-CTTCTTCCAGGAGATGAGAC-3′	57.3
HvCEN_F1	HvCEN	5′-AACTTTTCAGTTCAAGCTAGG-3′	54.0
HvCEN_F2	HvCEN	5′-ATCCATACCTGAGGGAGCACC-3′	61.8
HvCEN_F3	HvCEN	5′-TCCTTTTCATGCATGACTTGC-3′	55.9
HvCEN_R1	HvCEN	5′-TAAACTAGCTTGGGTTAGTGG-3′	55.9
HvCEN_R2	HvCEN	5′-AAATTAAGGATGGGGCCAATC-3′	55.9
HvCEN_R3	HvCEN	5′-AAGAGAAGAAGGGTATGGCTG-3′	57.9
HvELF3_delMariF1	HvELF3	5′-CTTCATCGTTTCAATTTTCTGCAG-3′	57.6
HvELF3_delMariF2	HvELF3	5′-ATTTTCTGCAGACAAGACAATGGG-3′	59.3
HvELF3_delMariR1	HvELF3	5′-CTGCACATCGTGATGTCCGGTTGA-3′	64.4
HvELF3_delMariR2	HvELF3	5′-CATCCCTCTGCACATCGTGATGTC-3′	64.4
Hvul_FT-R3	HvFT1	5′-GAAGGGGTCCAGCACG-3′	56.9
Hvul_FT-F3	HvFT1	5′-GATCCATCCATCGGTCTC-3′	56.0
Hvul_FT-F4	HvFT1	5′-ATCCGCTGGTTGTCGG-3′	54.3
Hvul_FT-F1	HvFT1	5′-GAATGTATCTACGATCAAGG-3′	53.2
Hvul_FT-F5	HvFT1	5′-TGATCATGATATGTGCATGC-3′	53.2
Hvul_FT-R5	HvFT1	5′-TAATGCTTAATTCGTGGCTGG-3′	55.9
Hvul_FT-R1	HvFT1	5′-CTGCGAGAATATATAAATGCC-3′	54.0
Hvul_FT-F2	HvFT1	5′-TGGAGCATCATTTCGTCC-3′	53.7
Hvul_FT-R4	HvFT1	5′-TAGGACTTGGAGCATCTGG-3′	56.7
Hvul_FT-R2	HvFT1	5′-TGGTTCAATCGCCAGAGC-3′	56.0
Hvul_FT-F2a	HvFT1	5′-TTATTTCAGCCCAGGGAC-3′	53.7
Hvul_FT-R2a	HvFT1	5′-TGTGGACGTGGTTCAATC-3′	53.7

### Bioinformatics Analysis

Sequencing results obtained were aligned to the relevant reference sequence and consensus sequences created for each gene for every variety sequenced using Geneious R11 (www.geneious.com). Open reading frames (ORFs) were translated using Geneious R11 and saved as a separate file. The resulting variety-specific consensus sequences, both nucleotide sequences and amino acid sequences, were compared to sequences publicly available in NCBI GenBank using multiple align features in Geneious R11 and haplotypes constructed manually. To identify potential significant effects on yield and heading for each of the four loci, BLUEs were compared using one-way analysis of variance (ANOVA) in Minitab® 19 (version 19.2020.1, Minitab LLC, State College, Pennsylvania, USA).

## Results

### Phenotypic Differences and Sequencing Results

**Figure 2:**
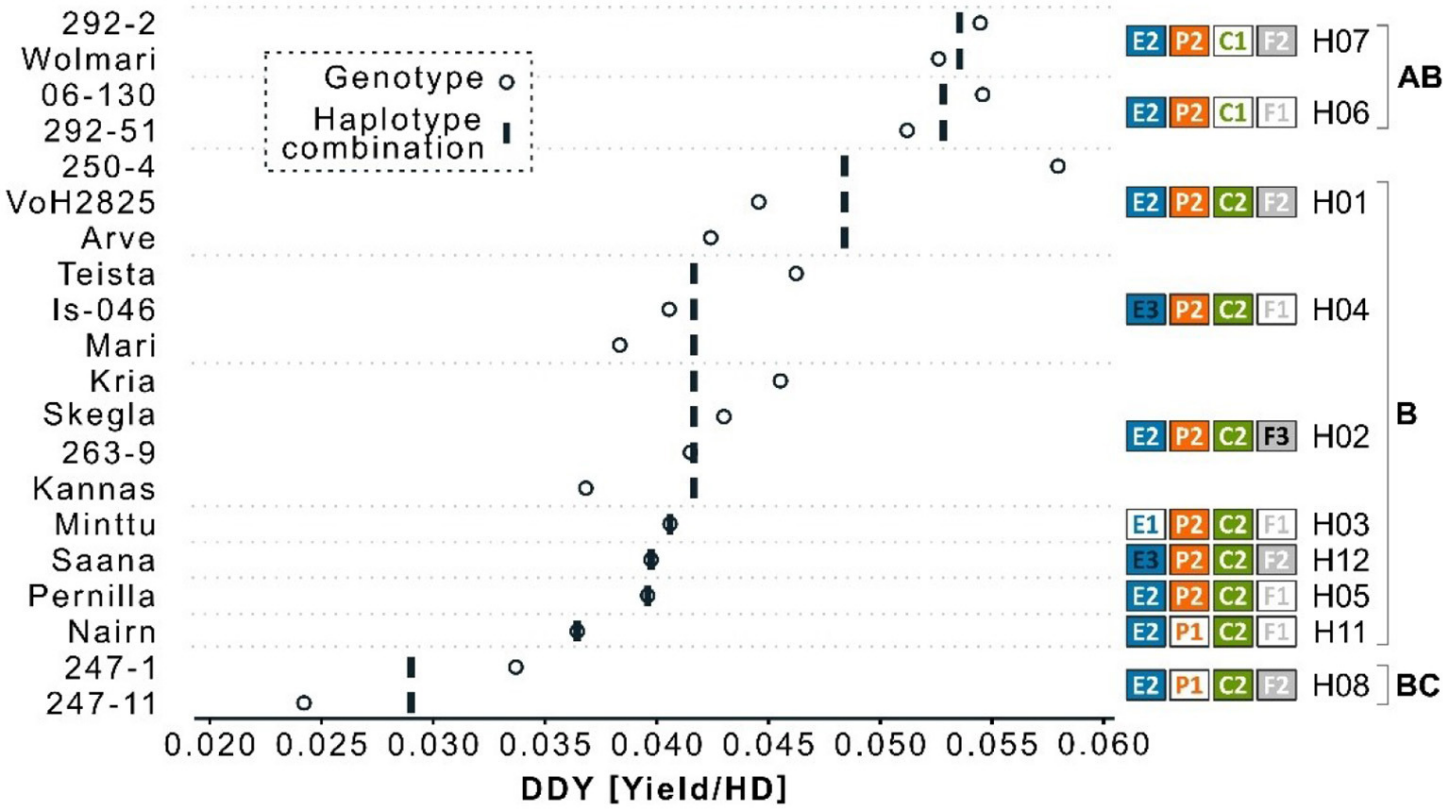
Index values of yield by heading day (DDY) for 20 barley genotypes. Open circles indicated DDY for each genotype. Vertical bands indicate the mean per haplotype combination. Haplotype combinations for the four flowering loci *HvELF3* (blue, E), *Ppd-H1* (orange, P), *HvCEN* (green, C), and *HvFT1* (gray, F) are shown to the right of the diagram. Tukey's test for significance resulted in a significant difference between haplotype H08 and H07/H06 (*p* = 0.03) and is indicated with the letters to the far right.

The best linear unbiased estimates for yield ranged from 1.61 to 4.43 t ha^−1^ and for heading from 66 to 83 days from sowing to heading (Figure 1). Three Icelandic breeding lines were extremely early with a heading day <70 days in the cold Icelandic climate. Two of them (“247-11” and “247-1”) were low yielding (1.61 and 2.28 t ha^−1^, respectively), whereas the third (“06-130”) yielded above average at 3.66 t ha^−1^. The DDY index ranged from 0.024 – 0.058 for the Icelandic breeding lines “247-11” and “250-4”, respectively (Figure 2). Despite the limited number of observations, there was a significant difference between the four genotypes with the highest DDY and the two genotypes with the lowest, respectively (*p* = 0.03).

Resequencing of the four genes, *HvELF3, Ppd-H1, HvCEN*, and *HvFT1* resulted in two to three allelic variants per gene with 12 haplotype combinations in the panel of Icelandic and north European genotypes (Table 1). Resequencing of the *HvELF3* gene identified two polymorphic sites giving rise to three haplotypes, referred to here as *HvELF3*^*E*1^, *HvELF3*^*E*2^, and *HvELF3*^*E*3^. The E2 allele is a single nucleotide polymorphism changing C in the reference sequence to a G and leading to a Glycine 316 to Alanine substitution (p.G316A), with the E3 allele including a 4 bp deletion in exon 2 (the so-called “Mari deletion”) in addition to the G to C SNP (Supplementary Figure 2). Among the 40 genotypes sequenced, 29 carried the allele *HvELF3*^*E*2^ and four lines carried the deletion allele found in the variety “Mari” (Table 1). There was no significant allele effect in *HvELF3* for either heading day (*p* = 0.11) or yield (*p* = 0.999) (Supplementary Tables 1, 2).

**Figure 3:**
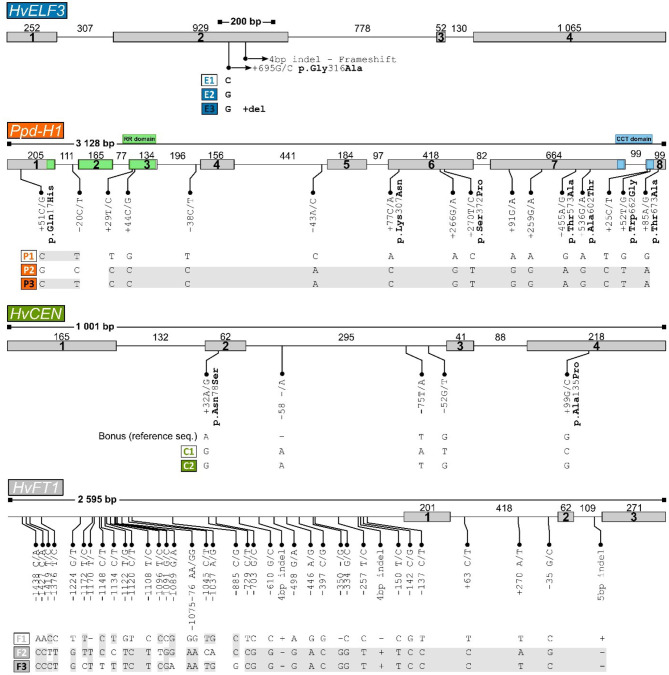
Structure of the four genes re-sequenced, *HvELF3, Ppd-H1, HvCEN*, and *HvFT1*, with polymorphisms constituting haplotypes identified. Exons are shown as boxes (size in base pairs below) and introns as lines (size in base-pairs above). The size of the region sequenced is shown in base pairs above the intron-exon structure with lines with filled boxes at each end. For each gene, the identified haplotypes are indicated below the corresponding line drawings. In the structure of the *HvELF3* (*Mat-a*) gene the location of the 4 bp “Mari” deletion is indicated with an arrow. Color coding of genes is the same in all figures.

Sequencing of the *Ppd-H1* gene in our panel identified 16 polymorphic sites, including 13 polymorphic sites in the coding regions of the gene, with the remaining polymorphisms in intronic regions (Figure 3). These polymorphisms lead to three haplotypes, referred to here as *Ppd-H1*^*P*1^*, Ppd-H1*^*P*2^, and *Ppd-H1*^*P*3^, with the P2 haplotype being the most frequent one which is found in 35 of the 40 lines sequenced. The *Ppd-H1*^*P*2^ allele is the previously reported mutated allele with seven missense mutations, which results in reduced photoperiod sensitivity commonly found in spring barley (Turner et al., 2005). Haplotypes P2 and P3 are only slightly different from each other with just a difference of two polymorphic sites, one found in intron 1 and the other being a missense polymorphism in exon 1 (p.Q17H) (Figure 3), with haplotype P3 only found in the variety “Golf” (Table 1). The *Ppd-H1*^*P*1^ allele described here corresponds to the wild type *Ppd-H1* allele, found primarily in winter barley and regulating flowering in response to increasing day length (Turner et al., 2005). There was a significant allele effect on yield (*p* = 0.00) between the two alleles P1 and P2 of *Ppd-H1* where yield data was available but no significant difference in heading day (*p* = 0.055) (Supplementary Tables 1, 2).

**Figure 4:**
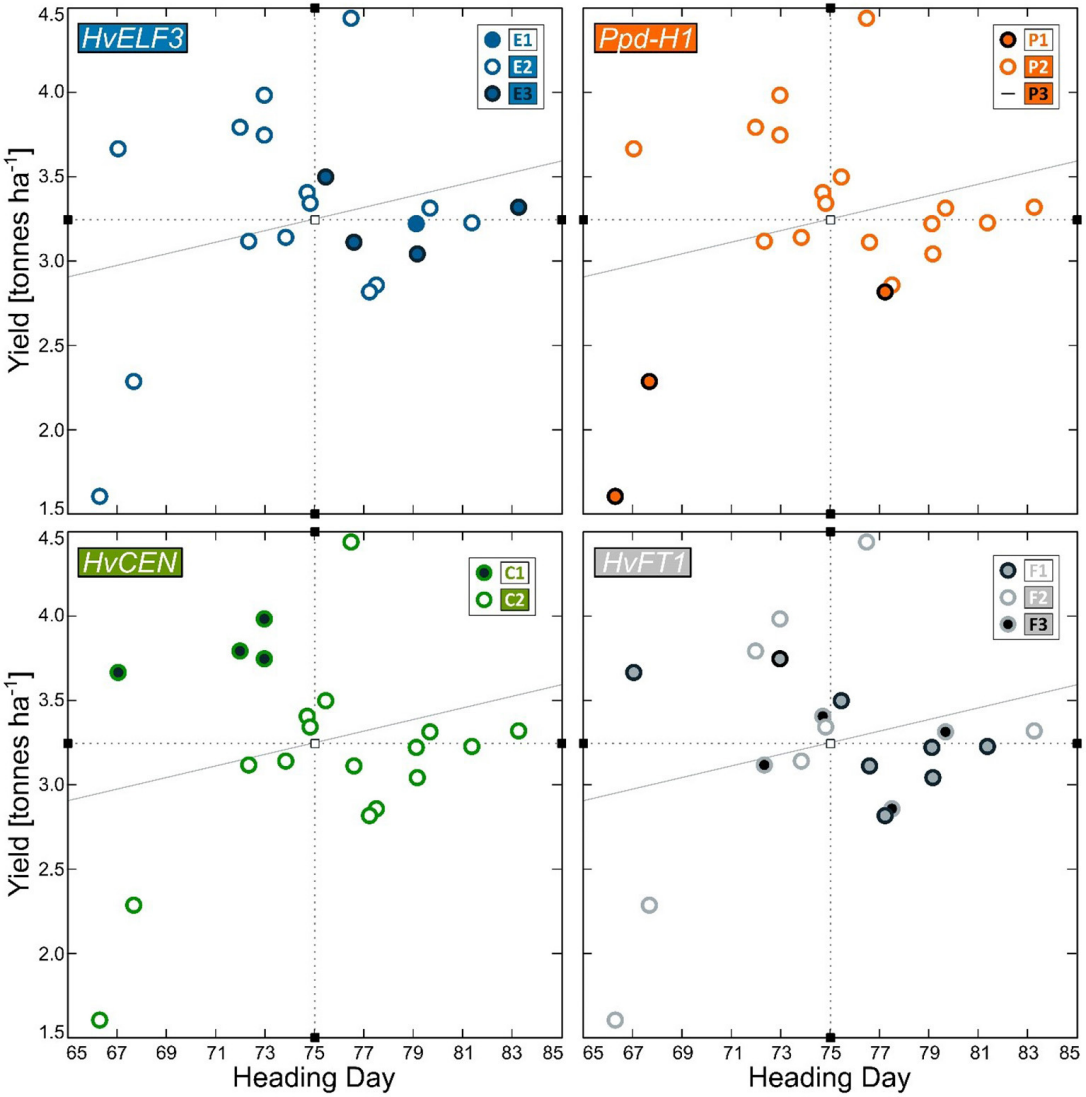
Heading day (days from sowing to heading) and Yield (t ha^−1^) for 20 barley genotypes grown in field trials in Iceland in the period 1987–2014. The four plots show results for the four re-sequenced genes *HvELF3* (blue), *Ppd-H1* (orange), *HvCEN* (green), and *HvFT1* (gray). The alleles are indicated with differently color-coded dots. Mean heading day and yield are indicated as dotted lines and the sloping gray line indicates the trendline.

Sequencing of the *HvCEN* gene identified three polymorphic sites in our collection, two additional polymorphisms in comparison to *cv*. Bonus the reference sequence. One of which is found in coding regions of the gene leading to an amino acid change (p.A135P), with three additional polymorphic sites found in intron 2 (Figure 3). These polymorphisms give rise to two haplotypes referred to here as *HvCEN*^*C*1^ and *HvCEN*^*C*2^, with the C2 haplotype being by far the most common haplotype, which is found in 35 of the 40 lines sequenced (Table 1). The C1 haplotype was found in the winter barley cultivar “Fimbul” and four other spring barley genotypes (Table 1), all of which combined earliness with high yield (Figure 4). There was a significant difference between the three alleles of *HvCEN* for yield (*p* = 0.036) but not for heading day (*p* = 0.064) (Supplementary Tables 1, 2).

Resequencing of the *HvFT1* gene resulted in 37 polymorphic sites, all of which are located outside the coding regions of the gene, with the vast majority found upstream of the first coding sequence (Figure 3). This leads to three different haplotypes, *HvFT1*^*F*1^, *HvFT1*^*F*2^, and *HvFT1*^*F*3^, with considerable overlap between haplotypes F2 and F3, and almost equal frequencies of haplotypes in our panel of lines (Table 1). There was no significant allele effect in *HvFT1* for either heading day (*p* = 0.466) or yield (*p* = 0.954) (Supplementary Tables 1, 2).

### Effect of Haplotypes on Yield and Heading

Intersection analysis of haplotypes and barley lines identified 12 haplotype combinations of the 4 genes with the *HvELF3*^*E*2^/*Ppd-H1*^*P*2^/*HvCEN*^*C*2^/*HvFT1*^*F*2^ (E2/P2/C2/F2) combination being the most frequent, found in 12 of the 40 lines sequenced, with frequencies of other haplotype combinations ranging from one to seven (Table 1). The two extremely early Icelandic lines sequenced, which are “247-1” and “247-11”, had the same haplotype composition, *HvELF3*^*E*2^/*Ppd-H1*^*P*1^/*HvCEN*^*C*2^/*HvFT1*^*F*2^ (see haplotype combination eight in Figure 2). The four early lines with high yielding capacity had the haplotype composition *HvELF3*^*E*2^*/Ppd-H1*^*P*2^*/HvCEN*^*C*1^ (Figure 2) differing only in *HvFT1*, with *HvFT1*^F1^ found in lines “06-130” and “292-51” and *HvFT1*^F2^ found in lines “292-2” and “Wolmari”. The *Pph-H1*^*P*1^ interacted with the *HvFT1*^*F*2^ allele, where the combination P1F2 had reduced yield (Supplementary Figure 3).

### Origin of Haplotypes in the Pedigree of Icelandic Lines

**Figure 5:**
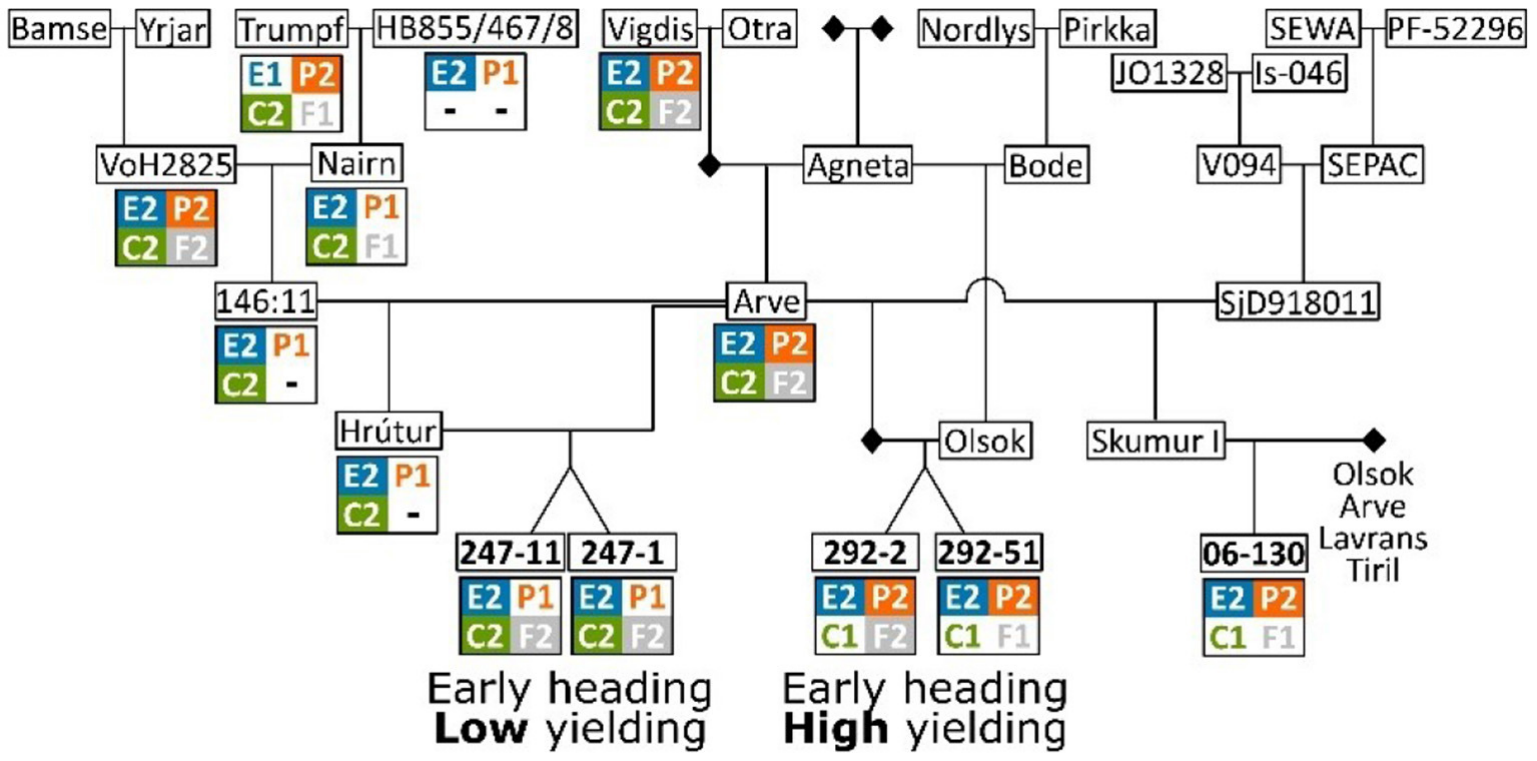
Pedigree of five Icelandic barley breeding lines: “247-1”, “247-11”, “292-2”, “292-51”, and “06-130”. Haplotypes for the four different genes re-sequenced are shown below each genotype. When possible, haplotypes are imputed for unsequenced genotypes. The full pedigree can be found in Supplementary Figure 1.

The pedigree of the Icelandic lines was reconstructed to understand the origin of the haplotype combinations found in the extremely early Icelandic lines, which are the low yielding sister lines “247-1” and “247-11” and the high yielding lines “06-130”, “292-2” and “292-51” (Figure 5; Supplementary Figure 1). This analysis shows that all the earliest genotypes are related to “Arve” but less related on the other side of the pedigree. While the *HvELF3*^*E*2^/*Ppd-H1*^*P*2^/*HvCEN*^*C*2^/*HvFT1*^*F*2^ haplotype combination is common in the pedigree for the two early Icelandic lines (see “Arve”, “VoH2825”, and “Vigdis” in Figure 5), the haplotype combination with *Ppd-H1*^*P*1^ (E2/P1/C2/F2 in Figure 5) is rare and is created with the haplotype inherited from “Nairn”.

The four early- and high-yielding lines were of a less certain origin. “Wolmari” is a cultivar from Finland; “06-130” is an Icelandic breeding line (a cross between “Skumur I” and one of the Norwegian cultivars “Arve”, “Olsok”, “Lavrans”, or “Tiril”); the sister lines “292-2” and “292-51”, are crosses between the combined genotype of “Arve” × “SjD918011” and “Olsok” (Figure 5).

## Discussion

Controlling the period from sowing to heading is important to maximize the utilization of the growing period and therefore has a direct effect on yield. Early heading has been correlated with early maturity (Göransson et al., 2019) and also acts to minimize the negative effect of adverse events at the end of the growing season, whether it be drought, heavy rains, frost, or extreme winds. The Icelandic barley breeding project, e.g., Hermannsson (1993) and Hilmarsson et al. (2017), has resulted in cultivars and breeding lines of interest due to increased earliness that could help us to better understand the control of flowering time in barley.

The main finding is the effect of the *HvCEN*^*C*1^ allele on DDY showing that earliness and high yield are possible to combine in a northern environment, which opens up possibilities to breed for earlier barley cultivars without losing yield. The spring type allele of *HvCEN*^*C*2^ has previously been reported to be virtually fixed in northern European spring barley (Tondelli et al., 2013; Fjellheim et al., 2014) and in earlier studies by Genome-Wide Association Studies (GWAS) of Nordic material, *HvCEN* has remained undetected, presumably due to a low allele frequency of alternate alleles in the studied populations (Göransson et al., 2019, 2021). The study of Fernandez-Calleja et al. (2021) reviewed the effect of diversity in *HvCEN* within three alleles, which are I, II, and III, whereof numbers II and III are of interest for this study. The type II (from “Nure”), corresponding to *HvCEN*^*C*1^, is predominantly found in winter barley and was in their study found to contribute to earliness and yield in the “Nure” × “Tremois” mapping population. The type III allele (from “Tremois”) is predominantly found in spring barley cultivars and is corresponding to *HvCEN*^*C*2^ in this study. Furthermore, the review by Fernandez-Calleja et al. (2021) reported latitudinal distribution of the two alleles in European barley cultivars indicating an adaptive role of the type III allele (*HvCEN*^*C*2^) in northern latitudes, originally reported in the studies of Comadran et al. (2012) and Bustos-Korts et al. (2019). In this study, we documented diversity in *HvCEN* in a panel of northerly adapted spring barley genotypes: three Icelandic genotypes and the Finnish spring barley cultivar “Wolmari” all carried the *HvCEN*^*C*1^ allele, which they also shared with the only winter barley included in the study, “Fimbul”. Moreover, all four spring barley genotypes carrying the winter-type *HvCEN*^*C*1^ combined earliness with a yielding capacity above average. This highlights its potential contribution to combine the two traits earliness and yield. There have been ambiguous reports on the role of *HvCEN* in yielding capacity as reviewed by the study of Fernandez-Calleja et al. (2021). The type III allele has the effect to delay flowering and has been advantageous in the cold and long growth seasons of northern Europe. The type II allele induces early flowering and is advantageous to the onset of flowering immediately after the south European winter months to enable the plant to develop seeds before the onset of the warm and dry summer. It is speculated in this study whether the type II allele can have an advantageous effect in the extreme environmental conditions of Iceland, where a delayed onset of flowering by type III allele may mean that the grain filling period will not coincide with the highest mean summer temperature peak at 11°C in July and August (Icelandic Meteorological Office, 2021). This would enable plants carrying the type II allele to initiate flowering early enough to make use of the highest summer temperatures for energy allocation to the kernels.

The four lines with significantly higher DDY (Figure 2) differed in *HvFT1*, a difference which we could not explain from the data in this study. The results are inconclusive for the role of *HvFT1* in both earliness and yield. In the study of Casas et al. (2011), they identified variation in the promoter and first intron of the *HvFT1* gene in a panel of Spanish landraces. The study conducted by Nitcher et al. (2013) reported an increase in copy number variation (CNV) at the *HvFT1* locus to be associated with early flowering in barley. They reported the CNV to originate from the Finnish spring barley variety “Tammi” (“Olli”/“Asplund”). Loscos et al. (2014) reported CNV of *HvFT1* in Nordic barleys, including Asplund, Maskin, and Tammi, carried a specific combination of only one promoter and several copies of exon 1. These lines all carry *HvFT1*^*F*2^ in this study and should reportedly be the earliest allele (Loscos et al., 2014). Although it cannot be concluded on CNV in *HvFT1* from this resequencing study, this is still an indication that the *HvFT1*^*F*2^ allele is contributing to the early flowering as was previously described by the CNV reported by the study of Nitcher et al. (2013). The study of Fernandez-Calleja et al. (2021) proposed a nomenclature of the *HvFT1* alleles based on an AG/TC SNP polymorphism in the first intron, which would group the F2 allele of this study as *VRN-H3a(T)*, and the F1 and F3 alleles would jointly be grouped as *vrn-H3c/d(n)*. More research is needed to elucidate the role of *HvFT1* in the genetic background of early Icelandic spring barley.

The two Icelandic lines“247-1” and “247-11” have shown special earliness in earlier studies (Göransson et al., 2019, 2021) and we show in this study that this is most likely due to a unique combination of haplotypes at four loci all known to play a role in flowering. This unique haplotype combination was created by crossing the Norwegian cultivar “Arve” with an Icelandic breeding line that was a cross between “Hrutur” × “Arve”, with the *Ppd-H1*^*P*1^ allele coming from “Nairn”, which is a Scottish variety. This variety seems to have inherited that haplotype from the variety “Fimbul”, a Swedish winter barley variety (Figure 5; Supplementary Figure 1). The winter-type allele *Ppd-H1*^*P*1^ is the wild-type and has been reported to advance flowering when day length increases. In nature, wild barley grows vegetatively during the wet winter months in the fertile crescent and is triggered by the increased day length in spring to initiate flowering before the summer heat and drought starts (Nevo et al., 2012). Most spring barley cultivars benefit from a delayed flowering in spring to establish vegetatively before transitioning to the reproductive stage, with the delay caused by the *Ppd-H1*^*P*2^ allele (Turner et al., 2005). The effect of the spring-type allele *Ppd-H1*^*P*2^ is well-studied in northern European barley, e.g., Turner et al. (2005) and Jones et al. (2008). We can now show that the winter-type contributes to extreme earliness among Icelandic spring barley genotypes. For the extremely early Icelandic barley lines we speculate that the *HvFT1*^*F*2^ from “Tammi” combined with the *Ppd-H1*^*P*1^ winter-type allele could be the reason for the extreme earliness of “247-11” and “247-1”. However, this comes with a severe yield penalty. The yielding capacity of the genotypes carrying the winter-type *Ppd-H1*^*P*1^ allele is well below average indicating a negative tradeoff between *Ppd-H1* ^*P*1^ derived earliness and yield. There is a noteworthy interaction between *Ppd-H1*^*P*1^ and *HvFT1*^*F*2^ with reduced yield indicating an interaction between the two genes (Supplementary Figure 5). The Scottish variety of barley “Nairn”, which carries the winter-type *Ppd-H1* ^*P*1^ allele without any effect on earliness or marked negative effect on yield, could here be benefitting from the haplotype combination of *Ppd-H1* ^*P*1^ and *HvFT1*^*F*1^. This combination was unique for “Nairn” and indicates an epistatic effect between the two genes (Supplementary Figure 5), with the P1F1 combination has little effect on yield, whereas the yield is reduced in the P1F2 combination (present in the genotypes “247-11” and “247-1”).

Allelic diversity at the *HvELF3* locus was less conclusive. *HvELF3* has previously been reported as the primary cause of adaptation to northern latitudes (Zakhrabekova et al., 2012). It can be concluded that the extreme earliness observed in “247-11”, “247-1”, and “06-130” is not caused by the Mari-deletion *HvELF*^*E*3^, which causes day length neutrality. It should be noted that the sequenced region is a region around exon 2, which leaves other previously described diverse regions such as exon 4 (Xia et al., 2017) unexplored.

The haplotypes with the highest and lowest DDY (Figure 2) were segregated in the two loci *Ppd-H1* and *HvCEN*. Genotypes carrying the day length sensitive allele *Ppd-H1*^*P*1^ in combination with *HvCEN*^*C*2^ were early heading, but low yielding. Genotypes carrying the *HvCEN*^*C*1^ allele combined with *Ppd-H1*^*P*2^ were both early heading and high yielding. This study speculates whether there is an interaction between the two loci, but the study did not include any *Ppd-H1*^*P*1^–*HvCEN*^*C*1^ haplotype, which would merit further studies. The allele *HvFT1*^*F*2^ seems to act epistatically to induce extreme earliness with winter-type alleles of both *Ppd-H1*^*P*1^ and *HvCEN*^*C*1^. In this study, we could not observe any haplotype combination of *HvFT1*^*F*2^/*Ppd-H1*^*P*1^/*HvCEN*^*C*1^. The number of identified alleles in the four loci gives a hypothetical number of 54 different haplotype combinations (3 × 3 × 2 × 3 = 54), whereas in the current panel we found 12. The small number of genotypes (20) with phenotype data was a limitation in the statistic analysis and data interpretation. In addition, we can conclude that there exist more known polymorphic sites for all genes than was found here (Supplementary Figure 3). This merits further studies that are underway using a multi-parent advanced generation intercross (MAGIC) approach, which has the potential to generate more recombinations.

Pyramiding of allelic combinations of flowering loci has the potential to induce earliness and further to combine earliness and yield capacity in spring barley. Gene-editing tools are now swiftly being developed, which have the potential to bypass past obstacles such as difficulty in breaking the linkage disequilibrium for loci located close to the centromeric regions. Better knowledge of epistatic effects of these genes with other loci in the flowering pathway such as *HvFT1* will enable breeders to fine-tune the flowering time of high yielding cultivars enabling a further expansion of cereal cultivation northwards.

## Data Availability Statement

The datasets presented in this study can be found in online repositories. The names of the repository/repositories and accession number(s) can be found at: https://www.ncbi.nlm.nih.gov/genbank/, MZ286789-MZ286948.

## Author Contributions

MG: conceptualization, data analysis, methodology, writing original draft, and review and editing. TS: genetic analyses, data analysis, and writing original draft. ML: methodology and review and editing. TB: data analysis and review and editing. JH: conceptualization, genetic analyses, data analysis, methodology, validation, writing original draft, and review and editing. All authors contributed to the article and approved the submitted version.

## Conflict of Interest

The authors declare that the research was conducted in the absence of any commercial or financial relationships that could be construed as a potential conflict of interest.

## Publisher's Note

All claims expressed in this article are solely those of the authors and do not necessarily represent those of their affiliated organizations, or those of the publisher, the editors and the reviewers. Any product that may be evaluated in this article, or claim that may be made by its manufacturer, is not guaranteed or endorsed by the publisher.
