# Supplementary material for: The Winter-Type Allele of HvCEN Is Associated With Earliness Without Severe Yield Penalty in Icelandic Spring Barley (Hordeum vulgare L.)
Source: Front Plant Sci. 2021 Sep 24;12:720238. doi: 10.3389/fpls.2021.720238 (PMC8500236; doi:10.3389/fpls.2021.720238)
Supplement: Supplementary file 1 [file Table_1.docx]

Supplementary Table S1. ANOVA output for heading day data.

**One-way ANOVA: HD versus HvELF3**

**Method**

| Null hypothesis | All means are equal |
| --- | --- |
| Alternative hypothesis | Not all means are equal |
| Significance level | α = 0,05 |

*Equal variances were assumed for the analysis.*

**Factor Information**

| **Factor** | **Levels** | **Values** |
| --- | --- | --- |
| HvELF3 | 3 | E1; E2; E3 |

**Analysis of Variance**

| **Source** | **DF** | **Adj SS** | **Adj MS** | **F-Value** | **P-Value** |
| --- | --- | --- | --- | --- | --- |
| HvELF3 | 2 | 91,52 | 45,76 | 2,52 | 0,110 |
| Error | 17 | 308,59 | 18,15 |  |  |
| Total | 19 | 400,11 |  |  |  |

**Model Summary**

| **S** | **R-sq** | **R-sq(adj)** | **R-sq(pred)** |
| --- | --- | --- | --- |
| 4,26056 | 22,87% | 13,80% | * |

**Means**

| **HvELF3** | **N** | **Mean** | **StDev** | **95% CI** |
| --- | --- | --- | --- | --- |
| E1 | 1 | 79,13 | * | (70,14; 88,12) |
| E2 | 15 | 73,79 | 4,41 | (71,47; 76,12) |
| E3 | 4 | 78,63 | 3,47 | (74,14; 83,12) |

*Pooled StDev = 4,26056*

**Tukey Pairwise Comparisons**

**Grouping Information Using the Tukey Method and 95% Confidence**

| **HvELF3** | **N** | **Mean** | **Grouping** |
| --- | --- | --- | --- |
| E1 | 1 | 79,13 | A |
| E3 | 4 | 78,63 | A |
| E2 | 15 | 73,79 | A |

*Means that do not share a letter are significantly different.*

**Tukey Simultaneous Tests for Differences of Means**

| **Difference of Levels** | **Difference of Means** | **SE of Difference** | **95% CI** | **T-Value** | **Adjusted P-Value** |
| --- | --- | --- | --- | --- | --- |
| E2 - E1 | -5,33 | 4,40 | (-16,63; 5,96) | -1,21 | 0,462 |
| E3 - E1 | -0,50 | 4,76 | (-12,73; 11,73) | -0,10 | 0,994 |
| E3 - E2 | 4,84 | 2,40 | (-1,32; 10,99) | 2,02 | 0,138 |

*Individual confidence level = 98,00%*

**One-way ANOVA: HD versus PpdH1**

**Method**

| Null hypothesis | All means are equal |
| --- | --- |
| Alternative hypothesis | Not all means are equal |
| Significance level | α = 0,05 |

*Equal variances were assumed for the analysis.*

**Factor Information**

| **Factor** | **Levels** | **Values** |
| --- | --- | --- |
| PpdH1 | 2 | P1; P2 |

**Analysis of Variance**

| **Source** | **DF** | **Adj SS** | **Adj MS** | **F-Value** | **P-Value** |
| --- | --- | --- | --- | --- | --- |
| PpdH1 | 1 | 75,66 | 75,66 | 4,20 | 0,055 |
| Error | 18 | 324,45 | 18,02 |  |  |
| Total | 19 | 400,11 |  |  |  |

**Model Summary**

| **S** | **R-sq** | **R-sq(adj)** | **R-sq(pred)** |
| --- | --- | --- | --- |
| 4,24555 | 18,91% | 14,41% | 0,00% |

**Means**

| **PpdH1** | **N** | **Mean** | **StDev** | **95% CI** |
| --- | --- | --- | --- | --- |
| P1 | 3 | 70,40 | 5,95 | (65,25; 75,55) |
| P2 | 17 | 75,845 | 3,982 | (73,682; 78,009) |

*Pooled StDev = 4,24555*

**Tukey Pairwise Comparisons**

**Grouping Information Using the Tukey Method and 95% Confidence**

| **PpdH1** | **N** | **Mean** | **Grouping** |
| --- | --- | --- | --- |
| P2 | 17 | 75,845 | A |
| P1 | 3 | 70,40 | A |

*Means that do not share a letter are significantly different.*

**Tukey Simultaneous Tests for Differences of Means**

| **Difference of Levels** | **Difference of Means** | **SE of Difference** | **95% CI** | **T-Value** | **Adjusted P-Value** |
| --- | --- | --- | --- | --- | --- |
| P2 - P1 | 5,45 | 2,66 | (-0,14; 11,03) | 2,05 | 0,055 |

*Individual confidence level = 95,00%*

**One-way ANOVA: HD versus HvCEN**

**Method**

| Null hypothesis | All means are equal |
| --- | --- |
| Alternative hypothesis | Not all means are equal |
| Significance level | α = 0,05 |

*Equal variances were assumed for the analysis.*

**Factor Information**

| **Factor** | **Levels** | **Values** |
| --- | --- | --- |
| HvCEN | 2 | C1; C2 |

**Analysis of Variance**

| **Source** | **DF** | **Adj SS** | **Adj MS** | **F-Value** | **P-Value** |
| --- | --- | --- | --- | --- | --- |
| HvCEN | 1 | 71,36 | 71,36 | 3,91 | 0,064 |
| Error | 18 | 328,75 | 18,26 |  |  |
| Total | 19 | 400,11 |  |  |  |

**Model Summary**

| **S** | **R-sq** | **R-sq(adj)** | **R-sq(pred)** |
| --- | --- | --- | --- |
| 4,27362 | 17,84% | 13,27% | 2,62% |

**Means**

| **HvCEN** | **N** | **Mean** | **StDev** | **95% CI** |
| --- | --- | --- | --- | --- |
| C1 | 4 | 71,25 | 2,85 | (66,76; 75,74) |
| C2 | 16 | 75,97 | 4,50 | (73,73; 78,22) |

*Pooled StDev = 4,27362*

**Tukey Pairwise Comparisons**

**Grouping Information Using the Tukey Method and 95% Confidence**

| **HvCEN** | **N** | **Mean** | **Grouping** |
| --- | --- | --- | --- |
| C2 | 16 | 75,97 | A |
| C1 | 4 | 71,25 | A |

*Means that do not share a letter are significantly different.*

**Tukey Simultaneous Tests for Differences of Means**

| **Difference of Levels** | **Difference of Means** | **SE of Difference** | **95% CI** | **T-Value** | **Adjusted P-Value** |
| --- | --- | --- | --- | --- | --- |
| C2 - C1 | 4,72 | 2,39 | (-0,30; 9,74) | 1,98 | 0,064 |

*Individual confidence level = 95,00%*

**One-way ANOVA: HD versus HvFT1**

**Method**

| Null hypothesis | All means are equal |
| --- | --- |
| Alternative hypothesis | Not all means are equal |
| Significance level | α = 0,05 |

*Equal variances were assumed for the analysis.*

**Factor Information**

| **Factor** | **Levels** | **Values** |
| --- | --- | --- |
| HvFT1 | 3 | F1; F2; F3 |

**Analysis of Variance**

| **Source** | **DF** | **Adj SS** | **Adj MS** | **F-Value** | **P-Value** |
| --- | --- | --- | --- | --- | --- |
| HvFT1 | 2 | 34,40 | 17,20 | 0,80 | 0,466 |
| Error | 17 | 365,71 | 21,51 |  |  |
| Total | 19 | 400,11 |  |  |  |

**Model Summary**

| **S** | **R-sq** | **R-sq(adj)** | **R-sq(pred)** |
| --- | --- | --- | --- |
| 4,63812 | 8,60% | 0,00% | 0,00% |

**Means**

| **HvFT1** | **N** | **Mean** | **StDev** | **95% CI** |
| --- | --- | --- | --- | --- |
| F1 | 8 | 76,12 | 4,47 | (72,66; 79,58) |
| F2 | 8 | 73,42 | 5,27 | (69,96; 76,88) |
| F3 | 4 | 76,05 | 3,21 | (71,16; 80,94) |

*Pooled StDev = 4,63812*

**Tukey Pairwise Comparisons**

**Grouping Information Using the Tukey Method and 95% Confidence**

| **HvFT1** | **N** | **Mean** | **Grouping** |
| --- | --- | --- | --- |
| F1 | 8 | 76,12 | A |
| F3 | 4 | 76,05 | A |
| F2 | 8 | 73,42 | A |

*Means that do not share a letter are significantly different.*

**Tukey Simultaneous Tests for Differences of Means**

| **Difference of Levels** | **Difference of Means** | **SE of Difference** | **95% CI** | **T-Value** | **Adjusted P-Value** |
| --- | --- | --- | --- | --- | --- |
| F2 - F1 | -2,70 | 2,32 | (-8,65; 3,25) | -1,17 | 0,489 |
| F3 - F1 | -0,08 | 2,84 | (-7,37; 7,21) | -0,03 | 1,000 |
| F3 - F2 | 2,63 | 2,84 | (-4,66; 9,92) | 0,92 | 0,633 |

*Individual confidence level = 98,00%*
