# Supplementary material for: The Winter-Type Allele of HvCEN Is Associated With Earliness Without Severe Yield Penalty in Icelandic Spring Barley (Hordeum vulgare L.)
Source: Front Plant Sci. 2021 Sep 24;12:720238. doi: 10.3389/fpls.2021.720238 (PMC8500236; doi:10.3389/fpls.2021.720238)
Supplement: Supplementary file 2 [file Table_2.docx]

Supplementary Table S2. ANOVA output for yield data.

**One-way ANOVA: Yield versus HvELF3**

**Method**

| Null hypothesis | All means are equal |
| --- | --- |
| Alternative hypothesis | Not all means are equal |
| Significance level | α = 0,05 |

*Equal variances were assumed for the analysis.*

**Factor Information**

| **Factor** | **Levels** | **Values** |
| --- | --- | --- |
| HvELF3 | 3 | E1; E2; E3 |

**Analysis of Variance**

| **Source** | **DF** | **Adj SS** | **Adj MS** | **F-Value** | **P-Value** |
| --- | --- | --- | --- | --- | --- |
| HvELF3 | 2 | 0,00103 | 0,000514 | 0,00 | 0,999 |
| Error | 17 | 6,80072 | 0,400043 |  |  |
| Total | 19 | 6,80175 |  |  |  |

**Model Summary**

| **S** | **R-sq** | **R-sq(adj)** | **R-sq(pred)** |
| --- | --- | --- | --- |
| 0,632489 | 0,02% | 0,00% | * |

**Means**

| **HvELF3** | **N** | **Mean** | **StDev** | **95% CI** |
| --- | --- | --- | --- | --- |
| E1 | 1 | 3,214 | * | (1,880; 4,549) |
| E2 | 15 | 3,245 | 0,690 | (2,901; 3,590) |
| E3 | 4 | 3,237 | 0,205 | (2,570; 3,904) |

*Pooled StDev = 0,632489*

**Tukey Pairwise Comparisons**

**Grouping Information Using the Tukey Method and 95% Confidence**

| **HvELF3** | **N** | **Mean** | **Grouping** |
| --- | --- | --- | --- |
| E2 | 15 | 3,245 | A |
| E3 | 4 | 3,237 | A |
| E1 | 1 | 3,214 | A |

*Means that do not share a letter are significantly different.*

**Tukey Simultaneous Tests for Differences of Means**

| **Difference of Levels** | **Difference of Means** | **SE of Difference** | **95% CI** | **T-Value** | **Adjusted P-Value** |
| --- | --- | --- | --- | --- | --- |
| E2 - E1 | 0,031 | 0,653 | (-1,646; 1,708) | 0,05 | 0,999 |
| E3 - E1 | 0,023 | 0,707 | (-1,792; 1,838) | 0,03 | 0,999 |
| E3 - E2 | -0,008 | 0,356 | (-0,922; 0,905) | -0,02 | 1,000 |

*Individual confidence level = 98,00%*

**One-way ANOVA: Yield versus PpdH1**

**Method**

| Null hypothesis | All means are equal |
| --- | --- |
| Alternative hypothesis | Not all means are equal |
| Significance level | α = 0,05 |

*Equal variances were assumed for the analysis.*

**Factor Information**

| **Factor** | **Levels** | **Values** |
| --- | --- | --- |
| PpdH1 | 2 | P1; P2 |

**Analysis of Variance**

| **Source** | **DF** | **Adj SS** | **Adj MS** | **F-Value** | **P-Value** |
| --- | --- | --- | --- | --- | --- |
| PpdH1 | 1 | 3,579 | 3,5790 | 19,99 | 0,000 |
| Error | 18 | 3,223 | 0,1790 |  |  |
| Total | 19 | 6,802 |  |  |  |

**Model Summary**

| **S** | **R-sq** | **R-sq(adj)** | **R-sq(pred)** |
| --- | --- | --- | --- |
| 0,423132 | 52,62% | 49,99% | 34,41% |

**Means**

| **PpdH1** | **N** | **Mean** | **StDev** | **95% CI** |
| --- | --- | --- | --- | --- |
| P1 | 3 | 2,235 | 0,606 | (1,722; 2,748) |
| P2 | 17 | 3,4199 | 0,3944 | (3,2043; 3,6355) |

*Pooled StDev = 0,423132*

**Tukey Pairwise Comparisons**

**Grouping Information Using the Tukey Method and 95% Confidence**

| **PpdH1** | **N** | **Mean** | **Grouping** | |
| --- | --- | --- | --- | --- |
| P2 | 17 | 3,4199 | A |  |
| P1 | 3 | 2,235 |  | B |

*Means that do not share a letter are significantly different.*

**Tukey Simultaneous Tests for Differences of Means**

| **Difference of Levels** | **Difference of Means** | **SE of Difference** | **95% CI** | **T-Value** | **Adjusted P-Value** |
| --- | --- | --- | --- | --- | --- |
| P2 - P1 | 1,185 | 0,265 | (0,628; 1,741) | 4,47 | 0,000 |

*Individual confidence level = 95,00%*

**One-way ANOVA: Yield versus HvCEN**

**Method**

| Null hypothesis | All means are equal |
| --- | --- |
| Alternative hypothesis | Not all means are equal |
| Significance level | α = 0,05 |

*Equal variances were assumed for the analysis.*

**Factor Information**

| **Factor** | **Levels** | **Values** |
| --- | --- | --- |
| HvCEN | 2 | C1; C2 |

**Analysis of Variance**

| **Source** | **DF** | **Adj SS** | **Adj MS** | **F-Value** | **P-Value** |
| --- | --- | --- | --- | --- | --- |
| HvCEN | 1 | 1,510 | 1,5104 | 5,14 | 0,036 |
| Error | 18 | 5,291 | 0,2940 |  |  |
| Total | 19 | 6,802 |  |  |  |

**Model Summary**

| **S** | **R-sq** | **R-sq(adj)** | **R-sq(pred)** |
| --- | --- | --- | --- |
| 0,542184 | 22,21% | 17,88% | 10,97% |

**Means**

| **HvCEN** | **N** | **Mean** | **StDev** | **95% CI** |
| --- | --- | --- | --- | --- |
| C1 | 4 | 3,7918 | 0,1349 | (3,2223; 4,3614) |
| C2 | 16 | 3,105 | 0,591 | (2,820; 3,390) |

*Pooled StDev = 0,542184*

**Tukey Pairwise Comparisons**

**Grouping Information Using the Tukey Method and 95% Confidence**

| **HvCEN** | **N** | **Mean** | **Grouping** | |
| --- | --- | --- | --- | --- |
| C1 | 4 | 3,7918 | A |  |
| C2 | 16 | 3,105 |  | B |

*Means that do not share a letter are significantly different.*

**Tukey Simultaneous Tests for Differences of Means**

| **Difference of Levels** | **Difference of Means** | **SE of Difference** | **95% CI** | **T-Value** | **Adjusted P-Value** |
| --- | --- | --- | --- | --- | --- |
| C2 - C1 | -0,687 | 0,303 | (-1,324; -0,050) | -2,27 | 0,036 |

*Individual confidence level = 95,00%*

**One-way ANOVA: Yield versus HvFT1**

**Method**

| Null hypothesis | All means are equal |
| --- | --- |
| Alternative hypothesis | Not all means are equal |
| Significance level | α = 0,05 |

*Equal variances were assumed for the analysis.*

**Factor Information**

| **Factor** | **Levels** | **Values** |
| --- | --- | --- |
| HvFT1 | 3 | F1; F2; F3 |

**Analysis of Variance**

| **Source** | **DF** | **Adj SS** | **Adj MS** | **F-Value** | **P-Value** |
| --- | --- | --- | --- | --- | --- |
| HvFT1 | 2 | 0,03719 | 0,01859 | 0,05 | 0,954 |
| Error | 17 | 6,76457 | 0,39792 |  |  |
| Total | 19 | 6,80175 |  |  |  |

**Model Summary**

| **S** | **R-sq** | **R-sq(adj)** | **R-sq(pred)** |
| --- | --- | --- | --- |
| 0,630806 | 0,55% | 0,00% | 0,00% |

**Means**

| **HvFT1** | **N** | **Mean** | **StDev** | **95% CI** |
| --- | --- | --- | --- | --- |
| F1 | 8 | 3,287 | 0,319 | (2,816; 3,757) |
| F2 | 8 | 3,234 | 0,916 | (2,763; 3,705) |
| F3 | 4 | 3,170 | 0,241 | (2,504; 3,835) |

*Pooled StDev = 0,630806*

**Tukey Pairwise Comparisons**

**Grouping Information Using the Tukey Method and 95% Confidence**

| **HvFT1** | **N** | **Mean** | **Grouping** |
| --- | --- | --- | --- |
| F1 | 8 | 3,287 | A |
| F2 | 8 | 3,234 | A |
| F3 | 4 | 3,170 | A |

*Means that do not share a letter are significantly different.*

**Tukey Simultaneous Tests for Differences of Means**

| **Difference of Levels** | **Difference of Means** | **SE of Difference** | **95% CI** | **T-Value** | **Adjusted P-Value** |
| --- | --- | --- | --- | --- | --- |
| F2 - F1 | -0,053 | 0,315 | (-0,862; 0,757) | -0,17 | 0,985 |
| F3 - F1 | -0,117 | 0,386 | (-1,108; 0,875) | -0,30 | 0,951 |
| F3 - F2 | -0,064 | 0,386 | (-1,056; 0,927) | -0,17 | 0,985 |

*Individual confidence level = 98,00%*
