# Supplementary material for: The Winter-Type Allele of HvCEN Is Associated With Earliness Without Severe Yield Penalty in Icelandic Spring Barley (Hordeum vulgare L.)
Source: Front Plant Sci. 2021 Sep 24;12:720238. doi: 10.3389/fpls.2021.720238 (PMC8500236; doi:10.3389/fpls.2021.720238)
Supplement: Supplementary file 3 [file Data_Sheet_1.docx]

**Figure S1 – S5.**

**Figure S1.**

**
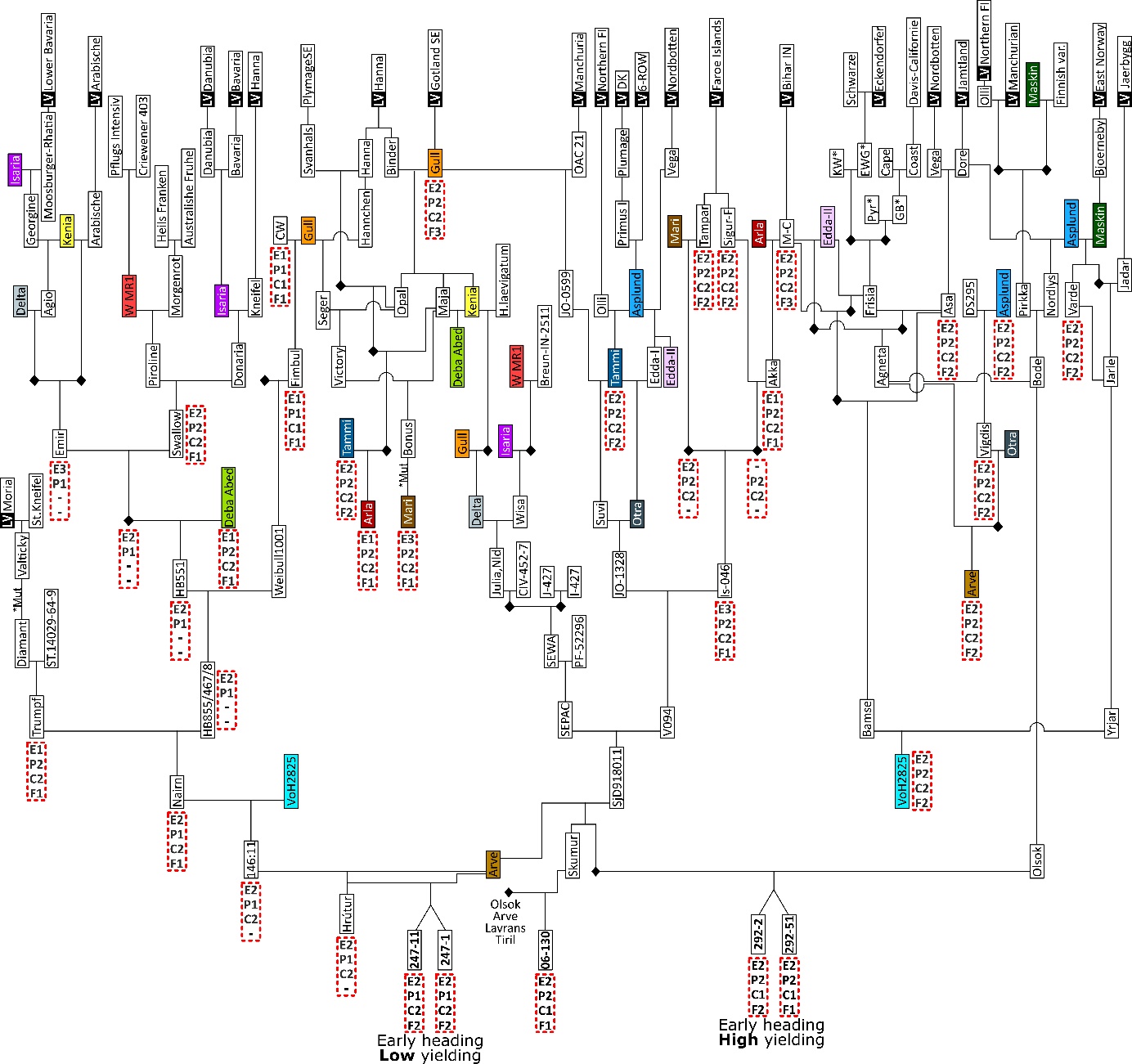
**

**Supplementary Figure S1. Pedigree of five Icelandic barley breeding lines: ‘247-1’, ‘247-11’, ‘292-2’, ‘292-51’, and ‘06-130’.** Abbreviated genotypes shown in the pedigree are indicated with an asterisk (*): G-B (Granat-Breustedt), K-W (Kalkreuther-WG), E-WG (Eckendorfer-WG), M-C (Monte-Christo), Pyr (Pyrthyjarven), and S-K (Starnovsky-Kneifel). Events of induced mutagenesis are marked “*Mut”.

**Figure S2.**

**
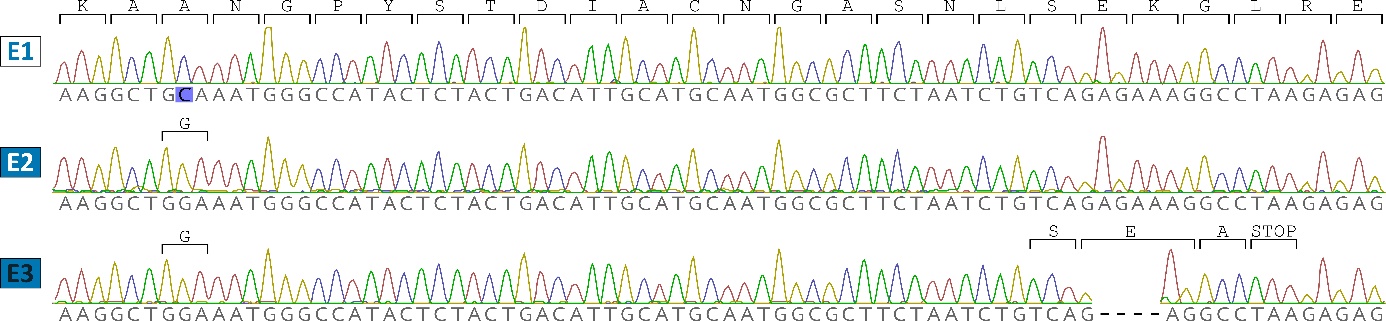
**

**Supplementary Figure S2.**  Resequencing of the *HvELF3* gene identified two polymorphic sites giving rise to three haplotypes, referred to here as *HvELF3^E1^*, *HvELF3^E2^*, and *HvELF3^E3^*. The E2 allele is a single nucleotide polymorphism changing C in the reference sequence to a G, and leading to a Glycine 316 to Alanine substitution (p.G316A), with the E3 allele including a 4 bp deletion in exon 2 (the so-called ‘Mari deletion’) in addition to the G to C SNP.

**Figure S3.**

**
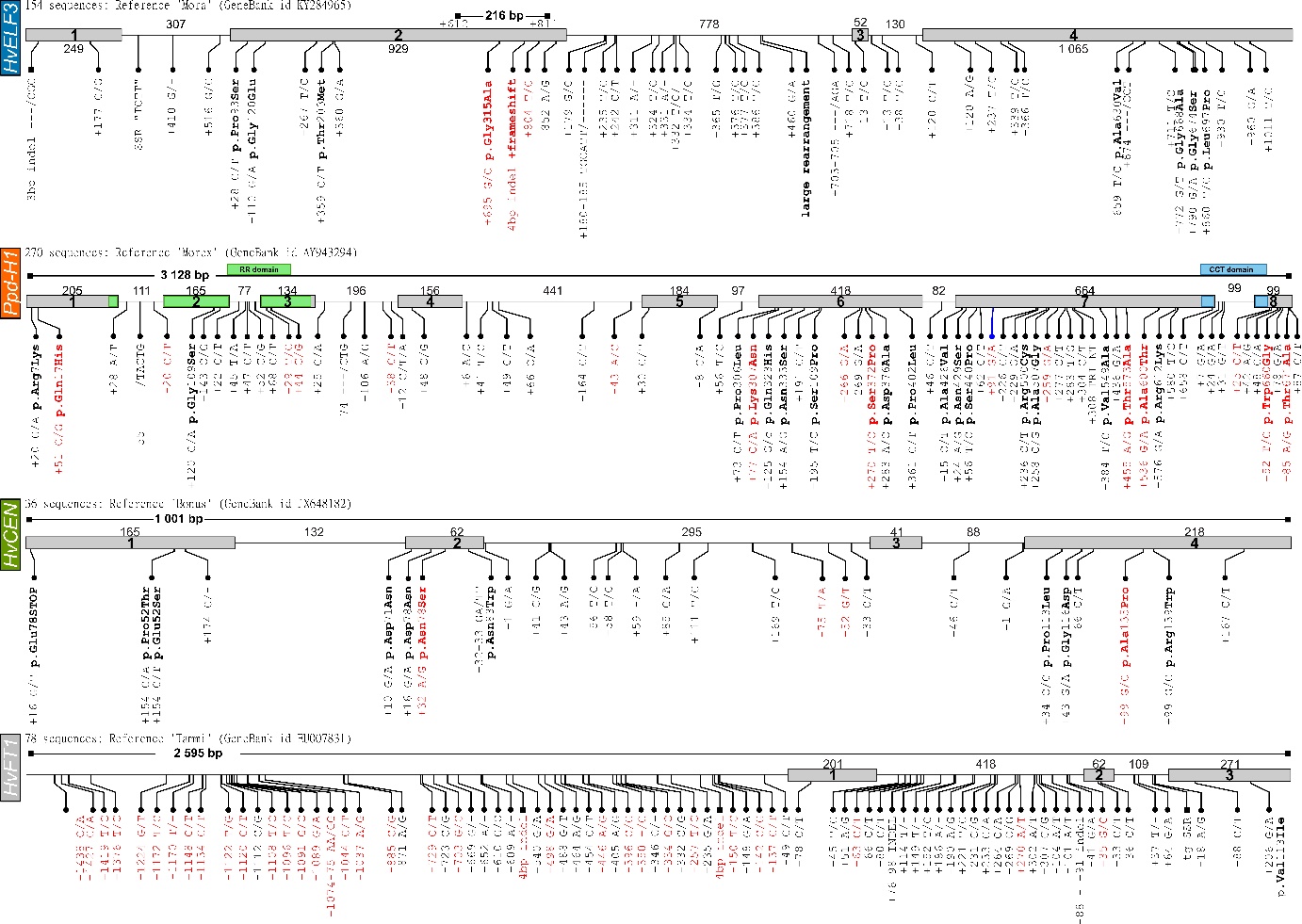
**

**Supplementary Figure S3. Detailed structure of the four genes re-sequenced, *HvELF3*, *Ppd-H1*, *HvCEN*, and *HvFT1*.** Exons shown as boxes (size in base-pairs below) and introns as lines (size in base-pairs above). The size of the region sequenced is shown in base-pairs above the intron-exon structure with lines with filled boxes at each end. Color coding of genes is the same in all figures. The polymorphic sites reported in this study are marked in red.

**Figure S4.**

**
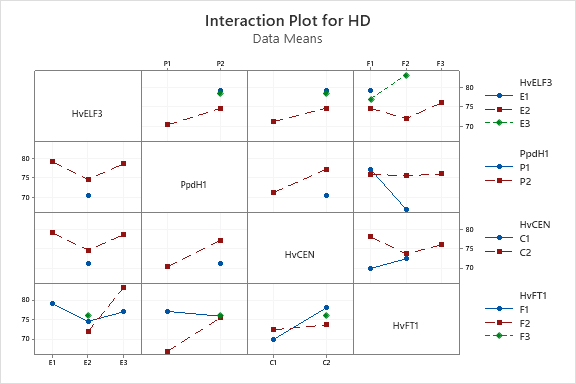
**

**Supplementary Figure S4. Interaction plot of heading day data and the allelic variation in four flowering genes.**

**Figure S5.**

**
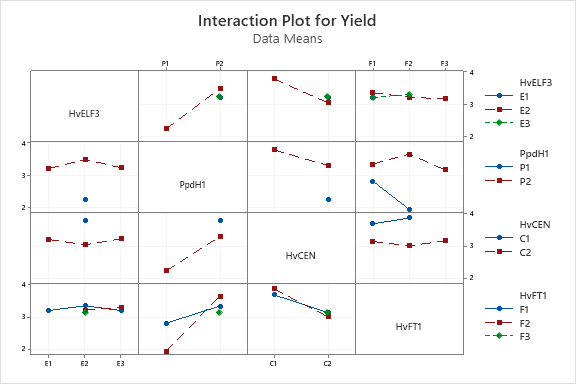
**

**Supplementary Figure S5. Interaction plot of yield data and the allelic variation in four flowering genes.**
