# Supplementary figures and images for: The Winter-Type Allele of HvCEN Is Associated With Earliness Without Severe Yield Penalty in Icelandic Spring Barley (Hordeum vulgare L.)
Source: Front Plant Sci. 2021 Sep 24;12:720238. doi: 10.3389/fpls.2021.720238 (PMC8500236; doi:10.3389/fpls.2021.720238)

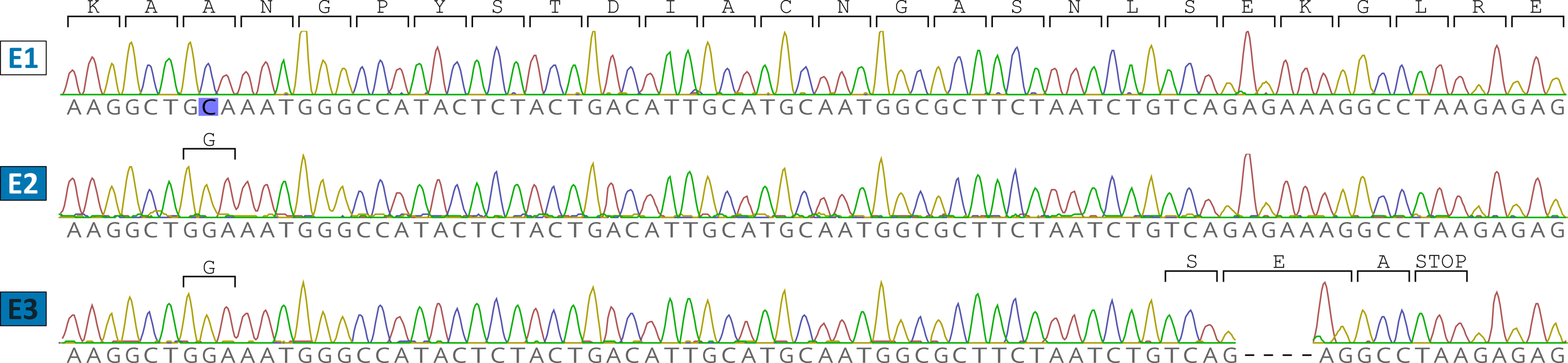

Supplement: Supplementary file 5 [file Image_2.JPEG]

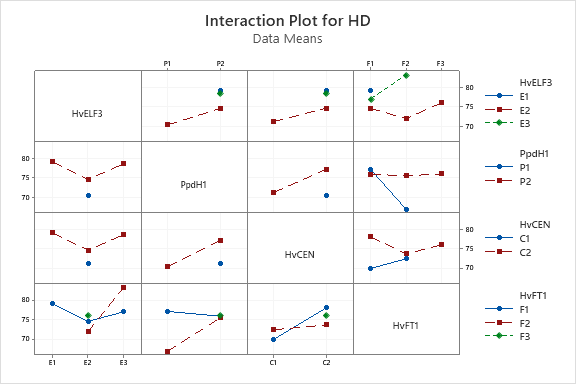

Supplement: Supplementary file 7 [file Image_4.TIF]

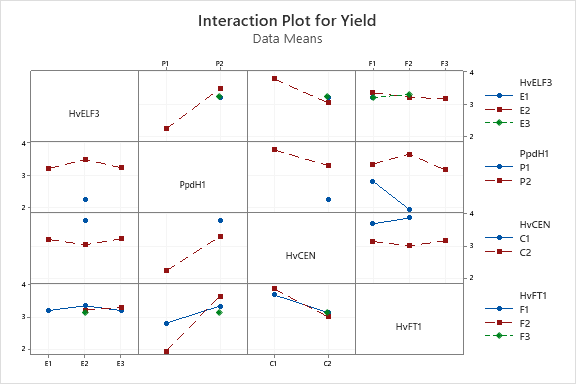

Supplement: Supplementary file 8 [file Image_5.TIF]
